# Editorial: Inflammation and Biomarkers in Osteoarthritis

**DOI:** 10.3389/fmed.2021.727700

**Published:** 2021-07-27

**Authors:** Ali Mobasheri, João Eurico Fonseca, Oreste Gualillo, Yves Henrotin, Raquel Largo, Gabriel Herrero-Beaumont, Francisco Airton Castro Rocha

**Affiliations:** ^1^Research Unit of Medical Imaging, Physics and Technology, Faculty of Medicine, University of Oulu, Oulu, Finland; ^2^Department of Regenerative Medicine, State Research Institute Centre for Innovative Medicine, Vilnius, Lithuania; ^3^Departments of Orthopedics, Rheumatology and Clinical Immunology, University Medical Center Utrecht, Utrecht, Netherlands; ^4^Department of Joint Surgery, First Affiliated Hospital of Sun Yat-sen University, Guangzhou, China; ^5^World Health Organization Collaborating Center for Public Health Aspects of Musculoskeletal Health and Aging, University of Liège, Liège, Belgium; ^6^Instituto de Medicina Molecular João Lobo Antunes, Faculdade de Medicina, Universidade de Lisboa, Lisbon, Portugal; ^7^Rheumatology Department, Hospital de Santa Maria, Centro Hospitalar Universitário Lisboa Norte (CHULN), Lisbon Academic Medical Centre, Lisbon, Portugal; ^8^SERGAS (Servizo Galego de Saude) and IDIS (Instituto de Investigación Sanitaria de Santiago), NEIRID Lab (Neuroendocrine Interactions in Rheumatology and Inflammatory Diseases), Research Laboratory 9, Santiago University Clinical Hospital, Santiago de Compostela, Spain; ^9^MusculoSKeletal Innovative Research Lab (mSKIL), Arthropole Liège, Department of Motricity Sciences, Center for Interdisciplinary Research on Medicines (CIRM), University of Liège, CHU Sart-Tilman, Liège, Belgium; ^10^Physical Therapy and Rehabilitation Department, Princess Paola Hospital, Marche-En-Famenne, Belgium; ^11^Bone and Joint Research Unit, Rheumatology Department, IIS-Fundación Jimenez Diaz UAM, Madrid, Spain; ^12^Department of Internal Medicine, Faculdade de Medicina da Universidade Federal do Ceará, Fortaleza, Brazil

**Keywords:** osteoarthritis, biomarkers, cartilage, synovium, inflammation

Osteoarthritis (OA) is the most common form of arthritis affecting more than 500 million people globally (1). It accounts for more pain and functional disability than any other musculoskeletal disease and is an important source of high societal and economic costs (2). Although the pathophysiology of OA is poorly understood (3), the risk factors associated with disease development are well-established. They include age (4), obesity (5), sex (6), previous incidence of joint injuries (7, 8), meniscal damage (9), joint instability (10), malalignment (11), genetics (12), bone shape (including anatomical deformities) (13), muscle weakness and sarcopenia (14), and metabolic disease (15–17). Although OA can affect any synovial joint, including joints in the hand, according to studies on the global burden of disease in 2010 (18) and 2017 (19), knee OA represents the greatest societal burden.

Beside mechanical derangement, inflammation plays a key role in the pathogenesis and progression of OA (20, 21). However, the inflammation associated with OA is not the same type and grade that is associated with rheumatoid arthritis (RA) and other inflammatory diseases of joints (22). It is becoming increasingly accepted that “low-grade” inflammation and the mechanisms that regulate it are relevant not only to joint pain and disability in OA (23), but also to joint trauma and the biomechanical damage sustained to joint tissues (24–26). Persistent synovitis as well as damage to the subchondral bone have been considered to play major roles in joint destruction, particularly in knee OA (27, 28). The association of meniscal damage with OA progression has highlighted the role of the meniscus and its biomechanical role in the joint (29–31). Therefore, the menisci may also participate in the inflammatory scenario of joints affected by OA (32).

Another important contributor to the process of “low-grade” inflammation in OA is the synovium (23, 33). There is evidence of cross-talk between articular cartilage, subchondral bone and synovium. Mechanistic evidence comes from *in vitro* and animal studies and clinical evidence from studies on patients with OA (34, 35). Synovial cells, particularly type A macrophage-like synoviocytes, are likely to be the major source of pro-inflammatory mediators within the joint (36). Moreover, there are differences in the profile of pro-inflammatory cytokine production in classically activated (M1) and alternatively activated (M2) macrophages (37, 38). Macrophage polarisation is an issue that may be relevant not only to emerging targeted therapies but also to ongoing efforts aimed at discriminating the different molecular endotypes and clinical phenotypes of OA (39, 40).

Biochemical markers (also called molecular markers, signature molecules or biomarkers) are biological molecules found in body fluids, or tissues that may be used as indicators of physiological and pathophysiological processes. They can be defined as “a characteristic that is objectively measured and evaluated as an indicator of normal biological processes, pathogenic processes, or pharmacologic responses to a therapeutic intervention.” (41). Biomarkers may be used to see how well patients respond to new treatments and interventions for a disease or condition. In OA biomarkers may be used to understand disease pathogenesis, study progression and define the molecular endotypes (42, 43). Biomarkers have been used very effectively to identify molecular endotypes and clinical phenotypes in other disease areas. For example, in asthma, biomarkers have been used to identify phenotypes and endotypes that characterise severe asthma (44, 45). However, in the field of OA we are lagging behind and need to catch up in order to enhance clinical trials and facilitate drug development. Biomarkers of early OA represent a major unmet need and more research needs to be done to identify biomarkers that characterise early events in the pathogenesis of OA.

The aim of this Research Topic was to assemble a comprehensive collection of authoritative articles focusing on fundamentals of the inflammatory scenario in OA joints and their relevance to existing and emerging biomarkers in this disease. One of the key priorities is the identification, characterisation and validation of biomarkers that define molecular endotypes of OA, serving as tools to discriminate different OA phenotypes.

MicroRNAs (miRNAs) are post-transcriptional regulators that are dysregulated in osteoarthritic tissues including the synovium. miRNAs are important contributors to OA synovial changes and to act as novel therapeutic targets. Tavallaee et al., reviewed the recently published literature investigating the roles that miRNAs play in OA-related synovial pathologies including inflammation, matrix deposition and cell proliferation. Their analysis of the literature has revealed that miRNAs contribute to synovial homeostasis, inflammation, fibrosis, angiogenesis, cell survival and cell apoptosis, contributing to OA synovial pathology.

The inflammation fuelled by metabolic imbalance, also known as “meta-inflammation,” is a type of chronic (long-lasting), persistent but “low-grade” systemic inflammation caused by multiple components involved in metabolic syndrome (MetS), including central obesity, adipokine dysregulation, and impaired glucose tolerance. Gratal et al., reviewed the literature focusing on purinergic regulation in OA cartilage and how different components of MetS modulate the purinergic system in OA. They described the critical role of receptors, such as adenosine A2A receptor (A2AR) and ATP P2X7 receptor in OA and assess how nucleotides regulate the inflammasome in OA.

Villalvilla et al. conducted an animal study using rabbits to investigate the effect of hypercholesterolemia induced by high-fat diet (HFD) in cartilage from OA rabbits, and how oxLDL affect human chondrocyte inflammatory and catabolic responses. They found that HFD intake does not modify cartilage structure or pro-inflammatory and catabolic gene expression and protein presence, both in healthy and OA animals. Their study concluded that dietary cholesterol intake may not be deleterious for articular cartilage but altered cholesterol metabolism may be involved in the associations observed in human disease.

Although biomarkers are important in OA research, clinical trials, and drug development, they have not yet had any significant impact on the clinical management of the OA and follow-up. Bernotiene et al., argued that emerging nano-technologies and immunoassay platforms that are already impacting on routine diagnostics and monitoring in other diseases could potentially serve as technological and strategic examples for enhanced clinical management of OA. Their review article explored the implementation of such technologies in OA research and therapy and discussed the challenges that hinder the development, testing, and implementation of new OA biochemical marker assays utilising emerging multiplexing technologies and biosensors.

Rajandran et al., evaluated the association between biomarkers of innate immunity and magnetic resonance imaging (MRI) features of early and late stages of knee OA. They investigated biomarkers of innate immunity associated with meniscal extrusion and synovial inflammation in earlier stage and bone marrow lesions (BMLs) in later stages of knee OA. They also observed associations between pro-inflammatory biomarkers and various MRI features in the early stages of knee OA. Their exploratory study supported the association between biomarkers of activated macrophages and synovial inflammation in the early stages of knee OA.

Lambert et al., reviewed the literature focusing on damage-associated molecular patterns (DAMPs) as biomarkers and potential therapeutic targets for OA. Their paper highlighted the central role of DAMPs in the interplay between immune responses and inflammation in OA.

Sun et al., used a rat model of OA to determine whether switching from an obesogenic diet to a normal chow diet can mitigate the detrimental effects of inflammatory pathways that contribute to OA pathology. Their results indicated that dietary switching from an obesogenic diet to a normal diet reduces body weight and restores metabolic parameters and suppresses synovial inflammation. They concluded that obesogenic diets induce systemic and synovial inflammation and dietary switching may be used as an intervention to slow down the progression of OA.

Work by de Melo Nunes et al. examined the chemical composition of glycosaminoglycans (GAGs) from normal and osteoarthritic cartilage and a reported reduced sulphur content in GAGs from OA patients, which is associated with a reduced zeta potential.

Finally, Zhang et al., reviewed the literature on synovial fibrosis in OA, establishing the concept that fibrosis is an eventual outcome of inflammation in OA. Therefore, new interventions are needed to slow the progression of fibrosis in OA and associated co-morbidities. They proposed the combined use of anti-fibrotic drugs with potential for therapy in OA.

We hope that you enjoyed reading these papers as much as we enjoyed editing them for this Research Topic in the rheumatology section of Frontiers in Medicine.

## Author Contributions

All authors contributed to the writing, editing, and revision of this editorial.

## Conflict of Interest

The authors declare that the research was conducted in the absence of any commercial or financial relationships that could be construed as a potential conflict of interest.

## Publisher's Note

All claims expressed in this article are solely those of the authors and do not necessarily represent those of their affiliated organizations, or those of the publisher, the editors and the reviewers. Any product that may be evaluated in this article, or claim that may be made by its manufacturer, is not guaranteed or endorsed by the publisher.
